# Pharmacokinetic study of the main components of Tanreqing capsules and Tanreqing injections in beagles by liquid chromatography–tandem mass spectrometry

**DOI:** 10.1186/s13020-022-00690-9

**Published:** 2022-12-05

**Authors:** Lili Cui, Liang Wang, Deduo Xu, Zhipeng Wang, Yong Chen, Xinhua Song, Fengjing Xu, Shouhong Gao, Lifeng Huang, Xia Tao, Wansheng Chen

**Affiliations:** 1 Department of Pharmacy, Second Affiliated Hospital of Naval Medical University, Shanghai, China; 2 Department of Pharmacy, Suzhou Science & Technology Town Hospital, Suzhou, China; 3 Suzhou Chien-Shiung Institute of Technology, Taicang, China

**Keywords:** Tanreqing capsule, Tanreqing injection, Pharmacokinetic parameters, LC–MS/MS, Beagles

## Abstract

**Background:**

Tanreqing capsules (TRQCs) and Tanreqing injections (TRQIs) are widely used in the treatment of respiratory diseases. In this study, a simple, rapid, and sensitive liquid chromatography–tandem mass spectrometry (LC–MS/MS) method was developed for simultaneous quantification of the main components of Tanreqing, which include chlorogenic acid, ursodeoxycholic acid, chenodeoxycholic acid, and baicalin, in beagle dog plasma to compare their pharmacokinetic parameters.

**Methods:**

Plasma samples were pretreated with protein precipitation. Chromatographic separation was performed on Waters Acquity UPLC HSS T_3_ (2.1 mm × 100 mm, 1.8 μm) column using a gradient elution with (A) 0.1% (v/v) formic acid aqueous solution and (B) acetonitrile. Six healthy beagles were divided into two groups, and a crossover, comparative pharmacokinetic study of TRQC (0.09 g/kg) and TRQI (0.5 mL/kg) after a single-dose administration or daily doses over 7 days was carried out. One group was administrated a single dose of TRQC and followed continuously for 7 days, whereas the other group was treated with TRQI in the same way.

**Results:**

The calibration curves were linear over the ranges of 2.00–1000.00 ng/mL for baicalin, 10.00–5000.00 ng/mL for ursodeoxycholic acid, 1.00–500.00 ng/mLfor chenodeoxycholic acid and chlorogenic acid, respectively. The relative standard deviation of both intra-day and inter-day accuracy is less than 11.23%. The average extraction recovery of all compounds was greater than 82.21%. The major pharmacokinetic parameters of the four compounds were not significantly different between the two formulations (P > 0.05).

**Conclusions:**

The measured levels of the four major components of TRQCs and TRQIs were comparable in these dogs, providing a reference for the clinical application of TRQCs instead of TRQIs.

**Supplementary Information:**

The online version contains supplementary material available at 10.1186/s13020-022-00690-9.

## Introduction

Guided by traditional Chinese medicine (TCM) theory, traditional Chinese medicine injections (TCMIs) are preparations of active substances extracted from single or combined Chinese or natural medicines using modern scientific techniques and methods [1]. According to the National Annual Report on Adverse Drug Reaction Monitoring of China (2017), the incidence rate of adverse reactions/events of TCMIs and oral preparations was 54.6% and 37.6%, respectively [2], and intravenous administration had resulted in more cases with severe adverse reactions. Due to a lack of comprehensive clinical data, the China Food and Drug Administration has restricted the clinical application of TCMIs [3]. Concerned with the safety of TCMIs, their manufacturers have turned to re-formulating these injections as capsules to avoid negative economic consequences and time costs during the injection re-evaluation process. Such changes in formulation have become an option for many companies. One example of this is the replacement of Shuanghuanglian Injection with Shuanghuanglian Oral Liquid [4, 5]. However, different drug formulations may have dramatically different drug pharmacokinetic patterns because of the in vivo disposal process.

Tanreqing is a traditional Chinese medicine compound preparation that comprises five herbs, Scutellaria baicalensis Georgi, Selenaretos thibetanus Cuvier, Saiga tatarica Linnaeus., Forsythia suspensa (Thunb.) Vahl and Lonicera japonica Thunb. It has significant anti-inflammatory, antiviral, antibacterial, and immunomodulatory activity, among other effects, and is widely used in the treatment of respiratory diseases [6, 7]. In our previous work, we analyzed the chemical components of TRQ preparation, and subsequently identified the major components such as baicalin, ursodeoxycholic acid, chenodeoxycholic acid, and chlorogenic acid, and pharmacokinetic studies were conducted for them [8, 9]. In recent years, Wang et al. [10] annotated 126 chemical components in Tanreqing injection, and He et al. [11] quantified the contents of baicalin, ursodeoxycholic acid, chenodeoxycholic acid, and chlorogenic acid by high performance liquid chromatography, and the four compounds were selected as main components for this studies. Baicalin shows characteristics associated with gastrointestinal hydrolysis, enterohepatic circulation, carrier transport, and complex metabolism, and can be converted into baicalein during the absorption process, the latter of which has antitumor, antibacterial, antioxidative, and some other pharmacological activities [12, 13]. Ursodeoxycholic acid and chenodeoxycholic acid have positive effects on enterohepatic circulation, and each can be converted into each other by intestinal flora [8, 14]. Chlorogenic acid has antipyretic, anti-inflammatory, antibacterial, antiviral, and other pharmacological activities [15, 16].

Since it first became commercially available in 2003, TRQI has been recommended by many clinical guidelines and treatment protocols. Since 2019, TRQI has been listed in the COVID-19 Diagnosis and Treatment Guidelines (Interim version 9, 8, 7, and 6) of China for the treatment of COVID-19 patients with critical symptoms [17, 18]. The ingredients and clinical applications of TRQIs and TRQCs are theoretically the same, but their routes of administration and dosages may affect their relative efficacy. TRQI is administered intravenously at 20 mL per day, whereas TRQC is administered orally with three capsules tid (three times per day). Although the in vivo processes of the four compounds have been reported [8, 19], these studies were all carried out in mice or rats. In addition, the reference value of the metabolic process data in the beagle breed of dogs is greater than that in rats. As the comparative pharmacokinetics of the main components of TRQI and TRQC are critical to the safe and effective replacement of the former by the latter, it is necessary to study and compare the pharmacokinetic parameters of the main components between TRQI and TRQC.

In this study, a two-cycle, crossover design was used to compare the key pharmacokinetic parameters between TRQC (0.09 g/kg) and TRQI (0.5 mL/kg) administered as a single dose, followed by seven consecutive days of administration in six healthy beagles. A LC–MS/MS method was developed to measure the levels of the main components of TRQC and TRQI in these dogs after treatment to provide a reference for the theoretical basis of replacement of TRQIs with TRQCs.

## Materials and methods

### Chemicals and reagents

All reference standards including baicalin (Lot: D0321AS), ursodeoxycholic acid (Lot: O0424AS), chenodeoxycholic acid (Lot: S0221AS), chlorogenic acid (Lot: N0805AS), and the internal standard (IS) puerarin (Lot: J1003AS) were provided by Dalian Meilun Biological Co., Ltd. (Dalian, China). Their purity is greater than or equal to 98%. HPLC-grade methanol and acetonitrile were provided by Merck Company (Darmstadt, Germany). Analytical-grade formic acid (≥ 95%) was purchased from McLean Biochemical Technology Co., Ltd. (Fairfield, USA). The water was double-distilled and was purchased from Watsons (Hong Kong, China). All other reagents used in the experiment were of analytical grade. TRQI (Lot: 1905101) and TRQC (Lot: 1907321) were provided by Shanghai Kaibao Pharmaceutical Co., Ltd. (Shanghai, China).

### LC–MS/MS instruments

An Agilent 1290-6460A ultra-high performance liquid chromatography–tandem mass spectrometry system, which includes the G4220A binary pump, G1969-80230 vacuum degasser, G4226A autosampler, and G1316C column oven, was used in our experiments, and the raw data acquirement and processing were carried out using MassHunter software (version B.06.00, Agilent Company, USA).

### Analytical conditions

Chromatographic separation of all four compounds was performed on Waters Acquity UPLC HSS T_3_ column (2.1 mm × 100 mm, 1.8 μm,; Waters, Milford, MA, USA) at a flow rate of 0.3 mL/min. The column temperature was kept at 35 °C, and the temperature in the autosampler was 4 °C. A gradient elution procedure was used with 0.1% formic acid aqueous solution as solvent A and acetonitrile as solvent B. The gradient elution consisted of 0–1 min, 10–55% B; 1–2.5 min, 55–70% B; 2.5–3.5 min, 70–95% B; 3.5–4.5 min, 95% B. The injection volume was 5 μL with a needle wash for 3 s using 5% methanol aqueous solution. The equilibration time between injections was 2 min.

Electrospray ionization (ESI) source and negative ionization mode were used for analyte ionization, and the data were collected in multiple reaction monitoring (MRM) mode. The ionization source parameters for MS were as follows: gas temperature, 350 °C; gas flow, 10 L/min; nebulizer, 40 psi; capillary, 4000 V; MS1 heater, 100 °C; MS2 heater, 100 °C. The optimized MRM conditions for all analytes are shown in Table 1.

**Table 1:** Optimized MRM parameters for the detection of analytes and IS

Analyte	Precursor ion(*m*/*z*)	Product ion(*m*/*z*)	Fragmentor (V)	Collision energy(eV)	Ionization mode	Chemical structure
Chlorogenic acid	353.1	191.0	70	10	Negative	
Ursodeoxycholic acid	391.3	391.3	250	0	Negative	
Chenodeoxycholic acid	391.3	391.3	250	0	Negative	
Baicalin	445.2	269.2	90	6	Negative	
Puerarin (IS)	414.9	266.9	145	24	Negative	

### Standard solution preparation

#### Preparation of stock solutions of compounds

Reference standards for chlorogenic acid, ursodeoxycholic acid, chenodeoxycholic acid, and baicalin were precisely weighed at 2.00 mg, 2.02 mg, 2.01 mg, and 2.02 mg, respectively, and were then dissolved using methanol to obtain stock solutions at 1.00 mg/mL, which were stored at – 80 °C. The stock solution of the IS (puerarin) was prepared and stored using the same method at a concentration of 1.00 mg/mL.

#### Preparation of working solutions and calibration standards

The stock solutions of all analytes were further mixed and diluted with methanol/water (10:90, v/v) solution to prepare the working solutions. An aliquot of 50 μL combined working solution was added to 450 μL blank plasma to obtain the following calibration standards: 1.00, 2.00, 5.00, 10.00, 50.00, 100.00, and 500.00 ng/mL for chlorogenic acid and chenodeoxycholic acid; 10.00, 20.00, 50.00, 100.00, 500.00, 1000.00, and 5000.00 ng/mL for ursodeoxycholic acid; and 2.00, 4.00, 10.00, 20.00, 100.00, 200.00, and 1000.00 ng/mL for baicalin. The quality control (QC) samples were prepared separately in the same way at 2.00, 10.00, 100.00 ng/mL of chlorogenic acid and chenodeoxycholic acid; 20.00, 100.00, 1000.00 ng/mL of ursodeoxycholic acid; and 4.00, 20.00, 200.00 ng/mL of baicalin. The QC samples were stored in a refrigerator at – 80 °C until retrieval (see Additional file 1).

### Sample pretreatment

100 μL plasma sample was drawn and transferred to 1.5 mL centrifuge tube prior to 300 μL protein precipitant (3:1 [v/v] methanol/acetonitrile, containing 0.1% formic acid and 100 ng/mL IS) was added, and then the sample was vortex-mixed for 3 min. The mixture was centrifuged at 20,000 ×*g* for 10 min, and 100 μL supernatant was transferred to the injection vial and analyzed directly by LC–MS/MS.

### Animals

This study was carried out in six beagles (three males and three females, 9.0–12.0 kg each), that were purchased from Junda Pet Hospital (Shanghai, China). This experimental protocol was approved by the Experimental Animal Ethics Committee of the Naval Military Medical University (Shanghai, China).

### Study of pharmacokinetics

#### Single-dose regimen

The six healthy beagles were divided into two groups: beagles 1–3 were all male and were assigned to the first group, and beagles 4–6 were all females and assigned to the second group. The two-cycle, crossover study was designed with a 7-day wash out period. For the single-administration process, the dogs in the first group were injected intravenously with 0.5 mL/kg TRQI (one-third of the normal daily dose), and blood samples were collected at 0, 5, 10, 15, 20, 30, and 45 min and then at 1, 2, 4, 6, 8, 12, and 24 h (a total of 14 time points) after injection. For each blood sample, 1 mL of blood was collected from the forelimb vein in heparin-treated tubes. The dogs in the second group were orally administrated 0.09 g/kg TRQC (one-third of the normal daily dose, and blood samples were collected in the same manner at 0, 10, 20, and 40 min and then at 1, 1.5, 2, 2.5, 3, 4, 6, 8, 12, and 24 h (a total of 14 time points). All blood samples were centrifuged at 4000 × *g* for 5 min, and each supernatant was collected and aliquoted. After a 7-day wash out period, the dogs in each group were administrated the other Tanreqing formulation in a crossover design, and blood samples were collected and processed in the same way.

#### Multiple-dose regimen

After the end of the single-dose experiment, the dogs in group one were given 0.5 mL/kg injection of TRQI once per day for 7 days following the wash out period, and the dogs in group two were given 0.09 g/kg TRQC once per day for 7 days**.** After the seventh administration, blood samples were collected at 0, 10, 20, and 40 min and then at 1, 1.5, 2, 2.5, 3, 4, 6, 8, 12, and 24 h (a total of 14 time points) and were processed as described in “Single-dose regimen” section. The resulting plasma samples were used to determine the steady-state plasma concentration of TRQI and TRQC. A crossover study was again carried out in both groups, as described in “Single-dose regimen” section, after a 7-day washing period.

#### Data analysis

All data obtained were analyzed using Drug and Statistics (DAS) 2.0 software (Chinese Society of Pharmacology), and a non-compartment analysis was used to calculate plasma pharmacokinetic parameters. The principal components of TRQI and TRQC were compared with an analysis of variance (ANOVA) and/or a two-tailed student-*t* test.

## Results and discussion

### Optimization of the LC–MS/MS conditions

To obtain better separation effects and higher sensitivity, we first optimized the chromatographic column, mobile phase, and mass spectrometry conditions and investigated the Agilent Zorbax SB-C_18_ column (2.1 mm × 150 mm, 3.5 μm), the Waters Xbridge SB-C_18_ column (2.1 mm × 50 mm, 3.5 μm), and the Waters Acquity UPLC HSS T_3_ column (2.1 mm × 100 mm, 1.8 μm), among others, for their ability. The HSS T_3_ column had better separation effects and a shorter analysis time. The analysis time was optimized from 15 to 10 min. Second, we tested the influence of different column temperatures on chromatographic separation and found that lower column temperatures resulted in a longer analysis time. The analysis time was optimized from 10 to 5 min, which shortens the analysis cycle [8]. Therefore, the column temperature was set at 35 °C, and the flow rate at 0.3 mL/min to ensure good separation effects. Third, the effects of ammonium acetate and formic acid in the mobile phase on the retention time and on the separation of the analytes were investigated. The aqueous phase containing 0.05%, 0.1%, or 0.2% formic acid and 2 mmol, 5 mmol, or 10 mmol ammonium acetate and their mixed aqueous solutions at different ratios were tested as the aqueous and organic phases**,** respectively. Water plus 0.1% (v/v) formic acid (phase A) and acetonitrile (phase B) resulted in the best peak shape and retention time.

The presence of endogenous isomers ursodeoxycholic acid and chenodeoxycholic acid in this experiment made the chromatographic separation of these two compounds difficult. In this study, based on their different retention times, we used their corresponding standards to identify their chromatographic peaks.

### Optimization of sample pretreatment

We first investigated different pretreatment methods of the biological samples, such as solid-phase extraction, protein precipitation, ultrafiltration, and so on. Solid-phase extraction and ultrafiltration were both affected by plastic adsorption, resulting in a low recovery (< 50%), and the matrix effects are strong and vary greatly (RSD% > 15%). For protein precipitation, we analyzed solvent mixtures containing methanol, acetonitrile, and different proportions of formic acid and ammonium hydroxide as additives. However, it is found that different ratios of additives have great impact on the experimental results. Ultimately, we found that precipitation with a mixture that included methanol and acetonitrile (3:1, v/v) plus 0.1% formic acid resulted in high extraction recovery and steady matrix effects, while other ratio of additives gave unsteady recovery and tailing peaks.

### Method validation

The methodology validation of this study was carried out in accordance with the guidance of the 2020 Chinese Pharmacopoeia. The validation criteria were based on previous reports [20, 21], including specificity, linearity, precision and accuracy, stability, extraction recovery, and matrix effects.

#### Specificity

**Fig. 1:**
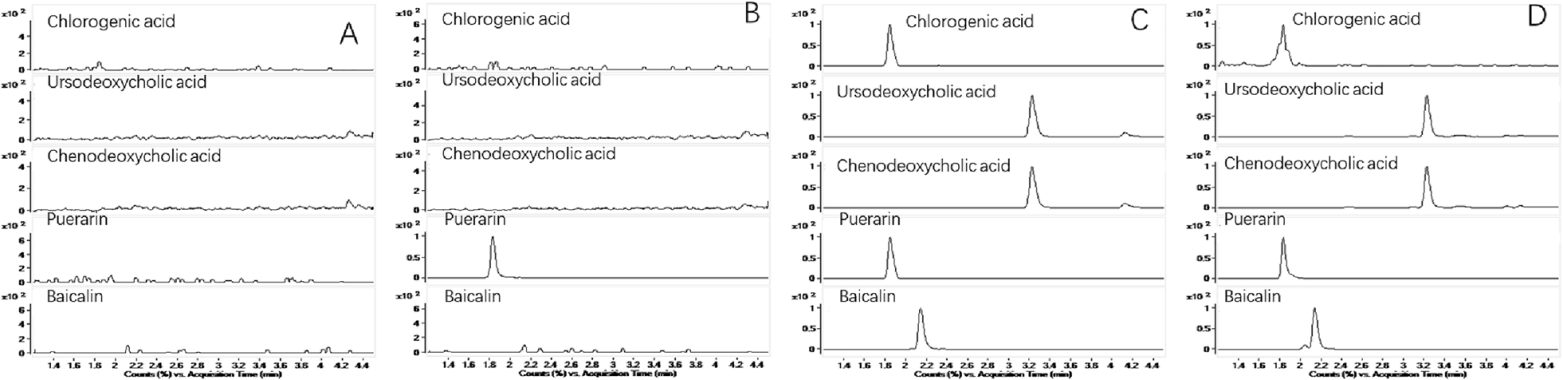
Typical MRM chromatograms showing the specificity of the LC–MS/MS method. **A** blank beagle plasma sample;** B** blank plasma sample spiked with 0.1 mg/mL puerarin (IS);** C** a spiked LLOQ sample; **D** a real sample

Specificity was assessed by comparing blank plasma samples from each of the six dogs, blank plasma spiked with IS, lower limit of quantification (LLOQ) sample, and real sample. We then examined the retention time of the chlorogenic acid, ursodeoxycholic acid, chenodeoxycholic acid, and baicalin, in addition to that of the IS, puerarin. There was no substantial interference in the retention time of the four analytes or IS (Fig. 1).

#### Linearity

The combined working solutions of the four compounds (chlorogenic acid, ursodeoxycholic acid, chenodeoxycholic acid, and baicalin) were diluted in blank plasma (1:9, v/v) to generate a series of calibration standards. The concentrations were 1.00, 2.00, 5.00, 10.00, 50.00, 100.00, and 500.00 ng/mL for chlorogenic acid and chenodeoxycholic acid; 10.00, 20.00, 50.00, 100.00, 500.00, 1000.00, and 5000.00 ng/mL for ursodeoxycholic acid; and 2.00, 4.00, 10.00, 20.00, 100.00, 200.00, and 1000.00 ng/mL for baicalin. The calibration standards were prepared in three replicates for each concentration, and LC–MS/MS analysis was carried out over 3 days. The best linearity and least-squares residuals for the calibration curves were achieved with a 1/*χ*^2^ weighting factor. The linear correlation coefficients were > 0.98 for all analytes. The deviations of the back-calculations of the seven calibration standards were all ± 15% (for LLOQ, ± 20%). The parameters of the four calibration curves are shown in Table 2.

**Table 2:** Linear regression equation, range, and correlation coefficient for the Tanreqing analytes in plasma samples

Analyte	Regression equation	LLOQ (ng/mL)	Calibration range (ng/mL)	*r*^2^
Baicalin	*y* = 0.002*x* + 1.021	2.00	2.00–1000.00	0.99
Ursodeoxycholic acid	*y* = 0.026*x* – 0.142	10.00	10.00–5000.00	0.99
Chenodeoxycholic acid	*y* = 0.038*x* − 0.020	1.00	1.00–500.00	0.99
Chlorogenic acid	*y* = 0.002*x* + 0.003	1.00	1.00–500.00	0.99

***x*** represents the measured concentration

***y*** represents the peak area ratio of compound to internal standard

#### Inter- and intra-day precision and accuracy

The QC samples and LLOQ samples were used to evaluate the inter- and intra-day precision and accuracy. These tests were carried out along with the linearity assessment with five replicates. The results showed good precision and accuracy, with intra-day and inter-day precision < 11.23% and accuracy within ± 14.00%. All data are summarized in Table 3.

**Table 3:** Inter- and intra-day precision and accuracy for the four analytes in dog plasma

Analyte	Nominal concentration (ng/mL)	Intra-day (n = 5)			Inter-day (n = 5)		
		Measured concentration (mean ± SD, ng/mL)	Precision (RSD%)	Accuracy (RE%)	Measured concentration (mean ± SD, ng/mL)	Precision (RSD%)	Accuracy (RE%)
Baicalin	2	2.00 ± 0.22	11.23	– 0.13	2.02 ± 0.20	9.71	0.78
	4	4.04 ± 0.17	4.10	0.99	4.01 ± 0.30	7.57	0.23
	20	18.77 ± 0.96	5.11	– 6.15	18.91 ± 1.21	6.42	– 5.44
	200	211.08 ± 3.43	1.63	5.54	212.40 ± 7.49	3.53	6.20
Ursodeoxycholic acid	10	10.90 ± 0.54	4.93	8.96	10.68 ± 0.52	4.85	6.83
	20	17.77 ± 0.38	2.13	– 11.17	17.46 ± 0.51	2.93	– 12.71
	100	87.02 ± 1.19	1.37	– 12.98	86.00 ± 2.16	2.51	– 14.00
	1000	1119.11 ± 26.08	2.33	11.91	1081.33 ± 53.16	4.92	8.13
Chenodeoxycholic acid	1	0.94 ± 0.04	4.10	– 5.63	0.99 ± 0.08	8.30	– 1.30
	2	1.98 ± 0.05	2.39	– 1.23	2.06 ± 0.10	4.70	3.00
	10	9.13 ± 0.36	3.98	– 8.71	9.74 ± 0.94	9.61	– 2.61
	100	94.07 ± 3.89	4.14	– 5.93	97.31 ± 4.01	4.12	– 2.69
Chlorogenic acid	1	0.97 ± 0.08	8.75	– 3.17	1.01 ± 0.08	7.77	1.46
	2	1.96 ± 0.15	7.77	– 2.07	2.11 ± 0.16	7.71	5.27
	10	9.49 ± 0.73	7.70	– 5.09	9.50 ± 0.91	9.54	– 5.01
	100	97.86 ± 1.82	1.86	– 2.14	100.09 ± 4.63	4.62	0.09

*RSD* Relative standard deviation; *RE* Relative error

#### Stability

The stability, which includes short-term stability (6 h at room temperature), long-term stability (− 80 °C for 30 days in the refrigerator), and freeze–thaw stability (three cycles from − 80 °C to room temperature), were assessed using QC samples at three concentration levels in three replicates. As shown in Table 4, three freeze–thaw cycles had no obvious effects on the analysis results of the QC samples. Similarly, there were no obvious effects on the analysis results of the samples with respect to short-term stability or long-term storage. In summary, the stability test showed satisfactory results under different conditions.

**Table 4:** Stability of four analytes in dog plasma (n = 3)

Analyte	Nominal concentration (ng/mL)	Short-term stability (6 h at room temperature)		Long-term stability (30 days at – 80 °C)		Freeze-thaw stability (3 cycles)	
		Mean ± S.D concentration (ng/mL)	RSD (%)	Mean ± S.D. concentration (ng/mL)	RSD (%)	Mean ± S.D. concentration (ng/mL)	RSD (%)
Baicalin	4	4.01 ± 0.31	7.75	3.50 ± 0.07	2.00	3.97 ± 0.36	9.12
	20	18.43 ± 0.75	4.05	17.47 ± 0.42	2.42	17.88 ± 0.92	5.13
	200	182.83 ± 7.72	4.22	175.01 ± 4.57	2.61	202.81 ± 10.87	5.36
Ursodeoxycholic acid	20	19.38 ± 0.97	5.01	18.77 ± 0.41	2.20	18.47 ± 1.66	9.00
	100	97.83 ± 0.62	0.63	90.80 ± 1.57	1.73	87.08 ± 1.95	2.24
	1000	1104.46 ± 16.75	1.52	1081.75 ± 20.70	1.91	1048.41 ± 75.10	7.16
Chenodeoxycholic acid	2	1.77 ± 0.06	3.21	1.92 ± 0.22	11.27	1.97 ± 0.17	8.89
	10	9.79 ± 0.78	7.94	10.10 ± 0.36	3.54	9.37 ± 0.95	10.16
	100	101.57 ± 0.54	0.53	101.23 ± 5.13	5.07	95.70 ± 4.66	4.87
Chlorogenic acid	2	1.98 ± 0.16	8.27	1.73 ± 0.05	3.00	2.07 ± 0.17	8.16
	10	8.97 ± 0.70	7.75	8.95 ± 0.42	4.65	10.03 ± 0.84	8.33
	100	88.28 ± 2.72	3.08	87.31 ± 4.62	5.30	95.65 ± 3.72	3.89

#### Extraction recovery and matrix effects

To examine the extraction recovery and the matrix effects, we used the high and low concentrations of QC samples to prepare six replicates at each of the two concentrations. The peak areas from the spiked samples were compared with the peak areas obtained from the spiked post-extraction samples at the same concentration, and the extraction recovery was determined. The matrix effects were calculated based on the ratio of the peak area of each spiked post-extraction sample to that of solvent-substituted sample at the same concentration. The results are shown in Table 5. The average extraction recoveries of baicalin, ursodeoxycholic acid, chenodeoxycholic acid and chlorogenic acid were between 82.21 and 99.17%. The average matrix effects for baicalin, ursodeoxycholic acid, chenodeoxycholic acid, and chlorogenic acid ranged from 85.14 to 112.81%.

**Table 5:** Extraction recovery and matrix effect data for the Tanreqing analytes in dog plasma (n = 6)

Analyte	Nominal concentration (ng/mL)	Extraction recovery		Matrix effect	
		Mean ± SD	RSD (%)	Mean ± SD	RSD (%)
Baicalin	4	82.21 ± 0.07	8.81	102.15 ± 0.14	13.72
	200	82.74 ± 0.05	6.45	93.41 ± 0.09	9.21
Ursodeoxycholic acid	20	86.42 ± 0.05	6.32	92.52 ± 0.07	7.82
	1000	98.05 ± 0.01	1.34	99.05 ± 0.02	1.52
Chenodeoxycholic acid	2	99.09 ± 0.09	9.25	112.81 ± 0.08	7.50
	100	99.17 ± 0.05	4.68	94.08 ± 0.03	3.06
Chlorogenic acid	2	94.62 ± 0.12	13.20	106.30 ± 0.10	9.35
	100	90.48 ± 0.05	5.90	85.14 ± 0.03	3.21

#### Application of this method in the pharmacokinetics analysis of the main components of TRQI and TRQC

We used this LC–MS/MS method to carry out a pharmacokinetic study of TRQC and TRQI and measure the concentrations of the four main components in plasma samples from the beagles. There was a huge difference in the administration route of TRQI and TRQC. As it is difficult for an individual to take nine capsules once a day, we first confirmed that a dose of three capsules of TRQC was comparable to one-third of a 20-mL injection of TRQI with respect to the main pharmacokinetic parameters. Based on the conversion ratio and pre-experimental validation (data not shown), the dose in this study was calculated as 0.5 mL/kg for TRQI and 0.09 g/kg for TRQC.

**Fig. 2:**
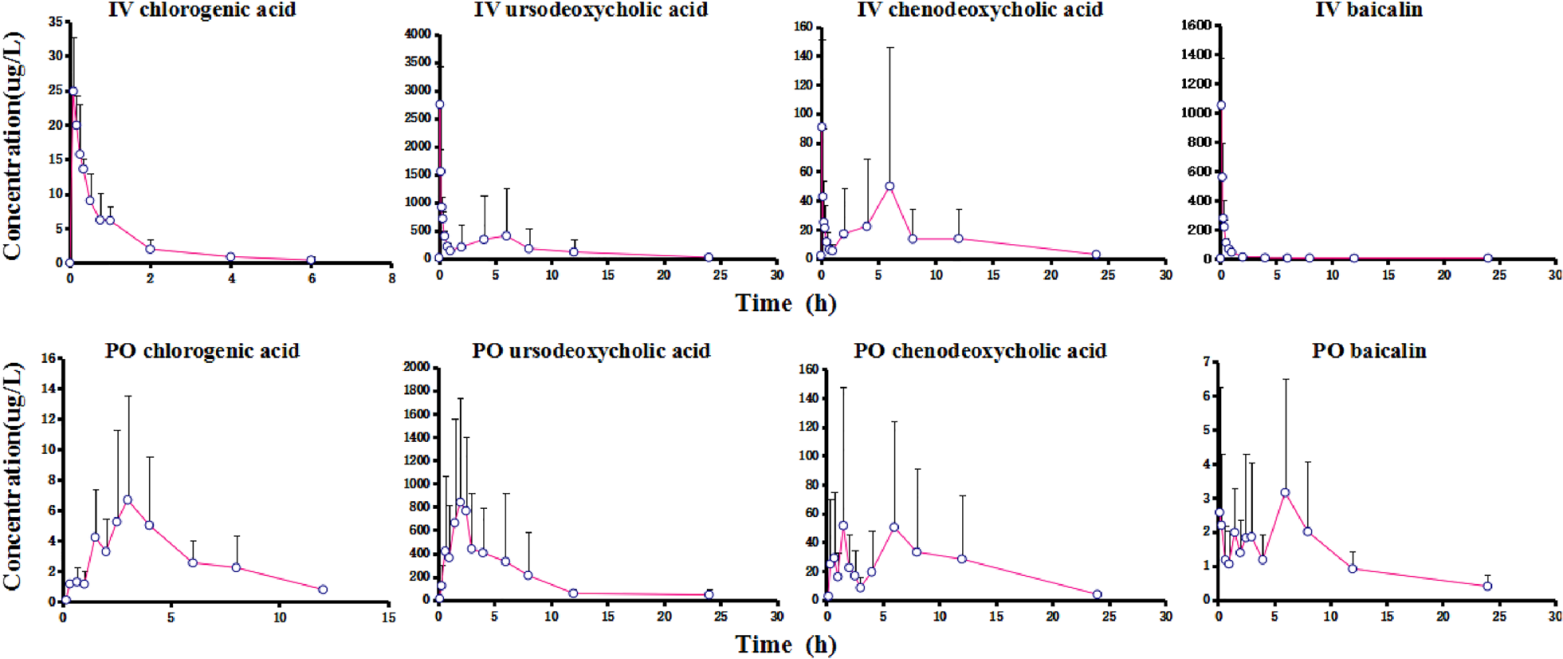
Mean plasma concentration of Tanreqing analytes over time after a single administration of TRQI or TRQC (n = 3). **a1**–**a4** Mean plasma concentrations of chlorogenic acid, ursodeoxycholic acid, chenodeoxycholic acid, and baicalin after a single dose of TRQI. **b1**–**b4** Mean plasma concentrations of chlorogenic acid, ursodeoxycholic acid, chenodeoxycholic acid, and baicalin after a single dose of TRQC

**Fig. 3:**
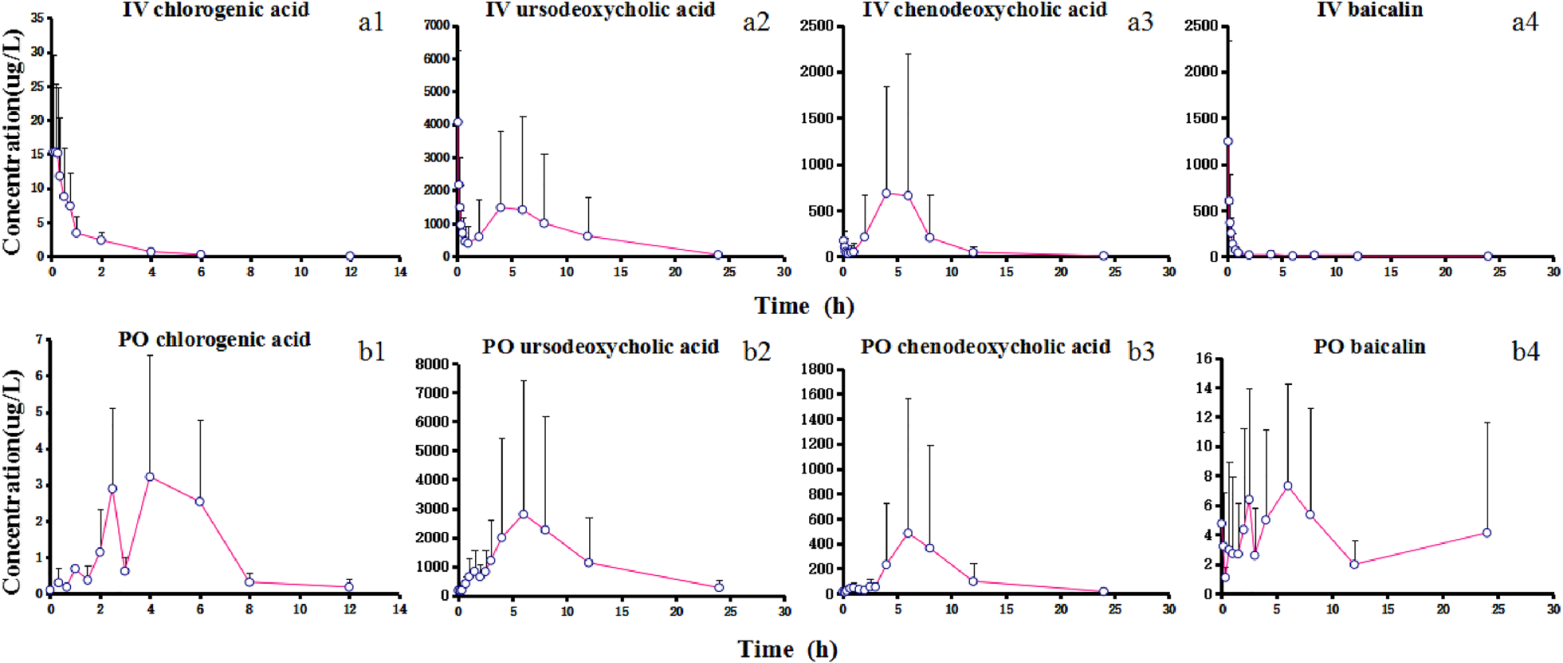
Mean plasma concentration of Tanreqing analytes over time after 7-day administrations of TRQI or TRQC (n = 3). **a1**–**a4** Mean plasma concentrations of chlorogenic acid, ursodeoxycholic acid, chenodeoxycholic acid, and baicalin after 7-day administrations of TRQI. **b1**–**b4** Mean plasma concentrations of chlorogenic acid, ursodeoxycholic acid, chenodeoxycholic acid, and baicalin after 7-day administrations of TRQC

The pharmacokinetic parameters of six healthy beagles after a single-dose intravenous injection of TRQI or a single-dose oral administration of TRQCs were estimated using a non-compartmental model (Tables 6 and 7). Results after 7 days of continuous dosing are shown in Table 7. The remaining pharmacokinetic parameters associated with TRQI and TRQC administration in this study are shown in Tables 8 and 9. The mean plasma concentration–time curves after a single administration and multiple administrations are shown in Figs. 2 and 3. The t_1/2_ values for ursodeoxycholic acid and chenodeoxycholic acid in another study were, respectively, 14 and 33 min [8], which are shorter than those of Hu’s [22] study (56 and 110 min, respectively), whereas the t_1/2_ value of baicalin was 216 min, four times longer than that previously reported (48 min). There are large differences in the results of these studies. Feng et al. [19] found that the t_1/2_ values among three doses of TRQI (3, 6, and 12 mL/kg) were not significantly different (p > 0.05). In addition, the area under the curve (AUC) values for baicalin were relatively high in that study, which demonstrated that TRQI and TRQC administration results in higher exposure levels of baicalin than the other components. Finally, the distribution and metabolism of baicalin in rat occurred relatively rapidly owing to a higher clearance rate (1.46 L/h/kg) and volume of distribution (1.07 L/kg) compared with the values for the three other components. In our study, the t_1/2_ values for TRQCs and TRQI were comparable for a single dose, and the T_max_ and C_max_ of TRQI were shorter and higher**,** respectively**,** than those of TRQC. We note that after dosing over a 7-day period, the t_1/2_ for all compounds in TRQC-treated dogs was approximately twice that of TRQI-treated dogs. Thus, TRQCs have a relatively longer half-life in vivo and may have an advantage with respect to an extended dosing interval. In summary, the exposure concentrations of TRQC and TRQI in vivo were roughly similar. In our study, there was no significant difference in the AUC_0–24 h_ and AUC_0–∞_ between the two formulations (P > 0.05). To the best of our knowledge, this is the first experiment in which the pharmacokinetic parameters of an injectable and capsule formulation from natural herbs have been compared. As both TRQIs and TRQCs had similar pharmacokinetic parameters, but TRQCs do not induce a severe adverse reaction**,** for the most part, as compared with TRQIs, TRQCs may be an effective alternative to TRQIs.

**Table 6:** Pharmacokinetic parameters of Tanreqing analytes in dog plasma after a single dose of TRQI or TRQC

Analyte	Parameter	Units	TRQI (0.5 mL/kg)		TRQC (0.09 g/kg)	
			Mean	SD	Mean	SD
Baicalin	t_1/2_	h	6.75	1.75	4.97	2.44
	T_max_	h	0.08	0.00	3.78	4.70
	C_max_	μg/L	1050.12	322.31	4.79	4.01
Ursodeoxycholic acid	t_1/2_	h	27.67	46.34	10.36	14.09
	T_max_	h	0.08	0.00	5.94	8.91
	C_max_	μg/L	2748.05	680.71	1332.75	801.89
Chenodeoxycholic acid	t_1/2_	h	5.67	3.81	5.93	4.87
	T_max_	h	1.07	2.42	7.53	9.16
	C_max_	μg/L	119.97	84.34	99.81	84.43
Chlorogenic acid	t_1/2_	h	0.89	0.30	1.45	0.39
	T_max_	h	0.13	0.07	3.75	2.90
	C_max_	μg/L	26.56	6.89	8.58	5.32

Samples were collected at 0, 5, 10, 15, 20, 30, and 45 min and then at 1, 2, 4, 6, 8, 12, and 24 h (a total of 14 time points) for TRQI, and 0, 10, 20, and 40 min and then at 1, 1.5, 2, 2.5, 3, 4, 6, 8, 12, and 24 h (a total of 14 time points) for TRIC

**Table 7:** Pharmacokinetic parameters of four analytes in dog plasma after administration of TRQI or TRQC for 7 days

Analyte	Parameter	Units	TRQI (0.5 mL/kg)		TRQC (0.09 g/kg)	
			Mean	SD	Mean	SD
Baicalin	t_1/2_	h	5.54	6.48	12.13	12.71
	T_max_	h	0.11	0.04	7.25	8.38
	C_max_	μg/L	1365.78	934.82	12.21	8.22
Ursodeoxycholic acid	t_1/2_	h	6.84	3.64	12.02	10.80
	T_max_	h	1.72	2.62	5.69	4.20
	C_max_	μg/L	5057.95	1592.37	3401.04	4312.06
Chenodeoxycholic acid	t_1/2_	h	6.25	6.25	13.73	21.22
	T_max_	h	1.72	2.62	4.72	4.29
	C_max_	μg/L	967.63	1446.30	520.63	1065.46
Chlorogenic acid	t_1/2_	h	0.88	0.31	1.56	1.04
	T_max_	h	0.18	0.08	3.17	0.93
	C_max_	μg/L	20.26	10.02	3.90	2.75

**Table 8:** The area under the curve (μg/L × h) after a single administration of TRQI and TRQCs in beagles

Treatment	No	Baicalin		Ursodeoxycholic acid		Chenodeoxycholic acid		Chlorogenic acid	
		AUC_0–24 h_	AUC_t–∞_	AUC_0–24 h_	AUC_t–∞_	AUC_0–24 h_	AUC_t–∞_	AUC_0–24 h_	AUC_t–∞_
TRQI (0.5 mL/kg)	1	44.51	57.67	18,217.56	18,383.85	1489.58	1513.91	29.65	30.35
	2	18.71	22.93	1101.92	1252.37	107.59	115.70	17.84	17.94
	3	0.47	0.07	2123.02	2129.29	238.29	238.42	23.30	23.38
	4	161.26	209.23	898.25	2922.46	89.03	143.43	11.17	12.10
	5	1.98	2.22	1164.19	1213.58	136.52	148.59	15.19	15.79
	6	39.93	54.11	992.23	1154.72	95.00	459.97	24.75	25.17
	Mean	44.40	57.71	4082.86	4509.38	359.34	436.67	20.32	20.79
TRQCs (0.09 g/kg)	1	16.04	17.28	1697.27	5384.48	104.10	212.35	21.05	93.50
	2	36.36	37.47	3189.09	3203.15	257.10	282.25	48.11	49.75
	3	11.82	16.12	4593.89	4594.24	421.35	421.42	33.19	36.26
	4	15.63	18.98	11,340.92	11,382.50	1330.27	1342.98	13.88	15.05
	5	1.15	1.19	1878.70	2503.16	126.03	147.57	-	-
	6	3.38	6.29	3702.57	4651.95	1077.47	80,865.10	-	-
	Mean	14.06	16.22	4400.40	5286.58	552.72	13,878.61	29.06	48.64
	*t*-Test	0.25	0.23	0.92	0.81	0.55	0.34	0.24	0.07

**Table 9:** The area under the curve (μg/L × h) after administration of TRQIs and TRQCs for 7 days in beagles

Treatment	No.	Baicalin		Ursodeoxycholic acid		Chenodeoxycholic acid		Chlorogenic acid	
		AUC_0–24 h_	AUC _0–∞_	AUC_0–24 h_	AUC _0–∞_	AUC_0–24 h_	AUC _0–∞_	AUC_0–24 h_	AUC _0–∞_
TRQI (0.5 mL/kg)	1	42.58	45.80	72,412.00	75,937.53	20,011.58	20,024.24	6.07	6.07
	2	369.81	533.81	1724.64	1859.36	120.94	183.39	5.24	5.59
	3	36.82	37.57	14,481.26	14,570.64	3241.10	3247.35	8.21	9.20
	4	527.34	669.59	4739.93	14,924.76	669.04	6190.14	19.08	19.29
	5	526.36	710.56	2560.78	4218.73	233.88	398.78	23.93	24.39
	6	865.34	1288.38	2665.15	2807.50	137.54	137.95	29.99	30.67
	Mean	394.71	547.62	16,430.63	19,053.09	4069.01	5030.31	15.42	15.87
TRQC (0.09 g/kg)	1	79.61	80.46	2762.49	2943.40	194.76	230.66	0.72	1.31
	2	149.00	164.07	23,894.62	24,369.21	1901.65	1928.12	3.19	3.20
	3	69.70	74.32	7185.97	7521.86	493.02	537.95	5.21	5.62
	4	68.04	88.26	109,038.51	112,128.46	16,729.10	16,833.54	22.55	23.20
	5	244.79	398.22	10,518.51	20,052.83	500.02	1963.93	11.37	13.34
	6	32.55	44.30	17,555.90	45,005.59	759.90	760.82	26.93	26.96
	Mean	107.27	141.61	28,492.67	35,336.89	3429.74	3709.17	11.66	12.27
	*t*-Test	0.06	0.07	0.56	0.44	0.88	0.75	0.55	0.57

The results of the study showed that the metabolism of baicalin in vivo was faster after intravenous injection of TRQI. In addition, ursodeoxycholic acid and chenodeoxycholic acid had obvious enterohepatic circulation. In mice, the metabolism of baicalin is slow, the metabolism of ursodeoxycholic acid and chenodeoxycholic acid is fast, and only chenodeoxycholic acid is associated with enterohepatic circulation. Thus these different results may reflect the different animals used in these studies. For TRQC in dogs, the oral availability of baicalin was small, which is consistent with a previous study [12]. However, the oral absorption of ursodeoxycholic acid and chenodeoxycholic acid reached a peak after 5 h and then decreased slowly in our study. Previous studies found that the absorption efficiency of chlorogenic acid analogs, neochlorogenic acid and isochlorogenic acid was low, and it was difficult to characterize their metabolic behavior [23, 24]^.^ However, the revelation of the metabolic behavior of chlorogenic acid in this study can provide certain reference value for the subsequent research.

## Conclusions

In this study, a rapid, sensitive, and convenient LC–MS/MS method was established and validated. This method has the advantages of a shorter running time and simpler pretreatment method and has been successfully applied to the quantification of four active components of Tanreqing in plasma samples from beagles. The exposure levels of the main components of TRQC and TRQI were largely consistent between both administration methods in these dogs. As compared with TRQI, TRQC resulted in a similar exposure among the beagles when the amount of prescription medicinal materials was increased four folds. TRQC may be an alternative for TRQI based on their comparable pharmacokinetics and lesser side effects of TRQC, although further study is needed to elucidate the clinical efficacy of TRQC as an alternative to TRQI.

## Supplementary Information

**Additional file 1:** **Table S1**. Optimization of sample pretreatment. **Table S2.** The optimization of mobile phase constitution. **Table S3.** Optimization of the LC–MS/MS conditions.

## Data Availability

The datasets used and/or analyzed during the current study are available from the corresponding author on reasonable request.
