# Supplementary material for: Pharmacokinetic study of the main components of Tanreqing capsules and Tanreqing injections in beagles by liquid chromatography–tandem mass spectrometry
Source: Chin Med. 2022 Dec 5;17:135. doi: 10.1186/s13020-022-00690-9 (PMC9721025; doi:10.1186/s13020-022-00690-9)
Supplement: Supplementary file 1 — Additional file 1: Table S1. Optimization of sample pretreatment. Table S2. The optimization of mobile phase constitution. Table S3. Optimization of the LC–MS/MS conditions. [file 13020_2022_690_MOESM1_ESM.docx]

**Table S1**. Optimization of sample pretreatment

| **Project** | **Conditions** | **Results** | |
| --- | --- | --- | --- |
|  |  | Extraction recovery | Matrix effect |
| Solid-phase extraction | | < 45% | > 150% |
| ultrafiltration | | < 40% | > 150% |
| Extraction solution | 50% methanol | Strong matrix effect,, poor response | |
|  | methanol | Strong matrix effect,, poor response | |
|  | 50% acetonitrile | Strong matrix effect,, poor response | |
|  | acetonitrile | Weak matrix effect，Peak splitting | |
|  | C：0.1% formic acid aqueous solution | Steady matrix effects，good peak type | |

**Table S2.** The optimization of mobile phase constitution

| **Project** | **Conditions** | **Results** |
| --- | --- | --- |
| Mobile phase | A1：0.05% formic acid aqueous solution | A1/B：Ion suppression, peak shape overbroad  A2/B：Weak ion suppression, good response  A3/B：Weak ion suppression, poor response  A4/B：Poor response  A4/B：Poor response  A4/B：Poor response |
|  | A2：0.1% formic acid aqueous solution |  |
|  | A3：0.2% formic acid aqueous solution |  |
|  | A4：2 mmol ammonium |  |
|  | A5：5 mmol ammonium |  |
|  | A6：10 mmol ammonium |  |
|  | B：acetonitrile |  |
|  | C：methanol | Weak elution force, bad peak shape, split, trailing, etc |

**Table S3.** Optimization of the LC-MS/MS conditions

| **Project** | **Conditions** | **Results** |
| --- | --- | --- |
| Column temperature | 25 °C | Retention time > 15 min |
|  | 30 °C | Retention time > 8 min |
|  | 35 °C | retention time = 4.5 min, good response |
|  | 40 °C | Poor response |
| Sprayer pressure | 35 psi | Poor response |
|  | 40 psi | Good response |
|  | 45 psi | Poor response |
|  | 50 psi | Poor response |
| Dry gas flow rate | 5 L/min | Very poor response |
|  | 8 L/min | Poor response |
|  | 10 L/min | Good response |
